# Genomics for Yield and Yield Components in Durum Wheat

**DOI:** 10.3390/plants12132571

**Published:** 2023-07-07

**Authors:** Francesca Taranto, Salvatore Esposito, Pasquale De Vita

**Affiliations:** 1 Institute of Biosciences and Bioresources (CNR-IBBR), 70126 Bari, Italy; francesca.taranto@ibbr.cnr.it; 2 Research Centre for Cereal and Industrial Crops (CREA-CI), CREA—Council for Agricultural Research and Economics, 71122 Foggia, Italy; salvatore.esposito@crea.gov.it

**Keywords:** quantitative trait loci, GWAS, genomic selection, candidate genes

## Abstract

In recent years, many efforts have been conducted to dissect the genetic basis of yield and yield components in durum wheat thanks to linkage mapping and genome-wide association studies. In this review, starting from the analysis of the genetic bases that regulate the expression of yield for developing new durum wheat varieties, we have highlighted how, currently, the reductionist approach, i.e., dissecting the yield into its individual components, does not seem capable of ensuring significant yield increases due to diminishing resources, land loss, and ongoing climate change. However, despite the identification of genes and/or chromosomal regions, controlling the grain yield in durum wheat is still a challenge, mainly due to the polyploidy level of this species. In the review, we underline that the next-generation sequencing (NGS) technologies coupled with improved wheat genome assembly and high-throughput genotyping platforms, as well as genome editing technology, will revolutionize plant breeding by providing a great opportunity to capture genetic variation that can be used in breeding programs. To date, genomic selection provides a valuable tool for modeling optimal allelic combinations across the whole genome that maximize the phenotypic potential of an individual under a given environment.

## 1. Introduction

**Figure 1:**
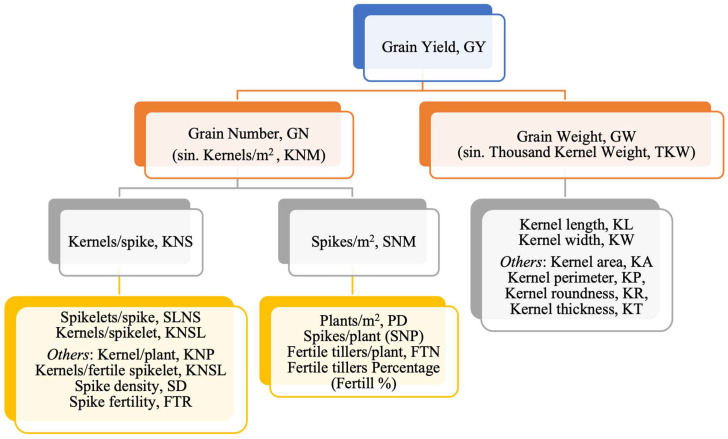
A reductionist approach to dissecting grain yield into its components.

Grain yield is the final expression of the multiple and complex individual physiological processes of a plant that has interacted with the climate and the environment during crop growing seasons. The occurrence of environmental stresses during stem elongation, flowering time, and grain-filling is harmful to durum wheat (Triticum turgidum subsp. durum), causing significant yield losses and negatively affecting the grain quality of raw material. In recent years, the durum wheat demand for pasta production has increased significantly globally [1]. Therefore, ensuring the supply for the pasta industry is an absolute priority, especially in the main production areas, particularly sensitive to climate change (i.e., Canada and the Mediterranean basin) [2]. In the areas vulnerable to environmental stresses and climate change, it is necessary to exploit the potential offered by modern technologies to understand the genetic, biochemical, and physiological mechanisms that regulate grain yield, and to develop a new generation of durum wheat varieties, more productive even in extreme environmental conditions [3]. In the last two decades, many efforts have been conducted to dissect the genetic basis of yield and yield components in durum wheat thanks to linkage mapping based on biparental populations and genome-wide association studies (GWAS) using DNA-based molecular markers, such as Amplified Fragment Length Polymorphism (AFLP), Simple Sequence Repeat (SSR), Diversity Array Technology (DArT), and Single Nucleotide Polymorphisms (SNPs) [4,5,6,7,8,9,10,11], and summarized by several recent reviews [12,13]. These studies led to the identification of hundreds of QTLs in different mapping populations with different types of molecular markers, with a limited impact on grain yield. Until now, due to the genetic complexity of yield in wheat, being controlled by a large number of genes with a small effect, a purely reductionist approach dissecting the grain yield (GY) into its individual components such as grain number (GN) [14] and grain weight (GW) [15,16], and its subcomponents [15,16,17], was adopted (Figure 1). The former is generated during the pre-anthesis period in wheat [18], whereas grain weight is defined after the anthesis as the result of the grain-filling duration and the grain-filling rate [19,20,21]. Specifically, GN is determined by the combination of several numerical subcomponents, including spikes/m^2^ (SNM) and kernels/spike (KNS) [22], whereas GW (also measured as thousand kernel weight, TKW) as well as grain size is determined mainly by kernel length (KL), kernel width (KW), and kernel thickness (KT) [23].

The effectiveness of this vision was demonstrated by the genetic gain achieved in terms of yield mainly due to the increase in the GN in both bread and durum wheat [24,25,26,27,28,29].

Unfortunately, compared to the continuous and steady genetic gain in yield achieved since the Green Revolution, the current annual increase in durum wheat yield is very slow, with a few rare exceptions. Probably because the relationships between the growth phenological stages and grain yield revealed a more complex pattern of relationships [30,31]. Indeed, several attempts to increase GY by increasing grain size in bread wheat have been hampered by the negative association with GN [32,33,34] and vice versa for durum wheat [14]. This indicates that, although GN and GW have been intensively studied, little is known about the genetic basis underlying this tradeoff in wheat, which thus remains a bottleneck for improving GY in wheat [33].

The rapid advances in next-generation sequencing (NGS) technologies coupled with improved wheat genome assembly and high-throughput genotyping platforms, as well as genome editing technology, is facilitating the identification of genes controlling critical agronomic traits such as yield and its yield components [35,36]. This means that in the post-genome era, for a complex genetic trait such as GY, controlled by multiple loci/genes and strongly influenced by the growing environment, a more appropriate holistic approach should be taken into account considering the whole wheat plant system, rather than the study of the individual yield components.

With respect to this premise, the review summarizes the genomic advances achieved in improving yield and its components as well as the potential impact of the reference wheat genome sequences will produce, in terms of new knowledge, in understanding the genetic mechanisms that regulate the tradeoff of yield components and/or the self-regulating capacity of the plant (i.e., plasticity) to face various environmental stresses.

## 2. Trends of Genomic Technologies to Advance Yield and Yield Components in Durum Wheat

**Figure 2:**
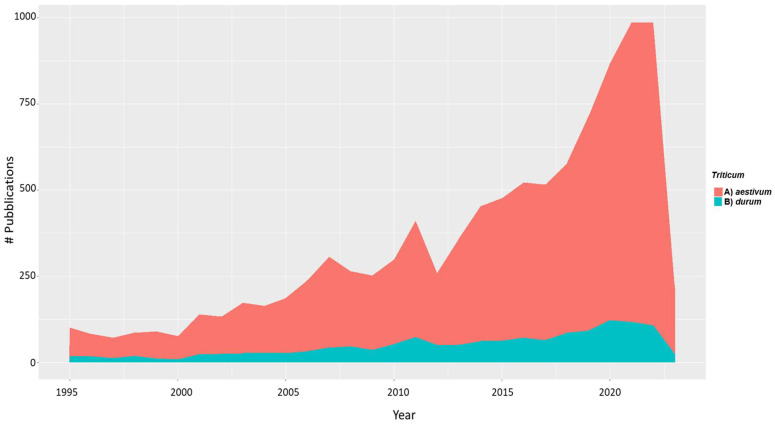
Number of papers in which “yield” and “yield components” traits were investigated by using genomic approaches in wheat from 1995 to 2021. (A) complete query = (“bread” or “aestivum”) AND “yield” AND (“genetic” or “genomic); (B) complete query = (“durum wheat”) AND “yield” AND (“genetic” or “genomic”). The figure was drawn with ggplot2 library [39].

For measuring the development trends in the use of genomic approaches to study yield and yield components in durum wheat, Figure 2 shows the number of academic papers published from 1995 to date. The research was based on the information available in the Web of Science database (www.webofknowledge.com, 30 May 2022), field “Topic”, category “Plant science” and “Agriculture”. Different keywords (i.e., “yield”, “QTL (Quantitative Trait Loci) mapping”, “GWAS (Genome Wide Association Studies)”, “genomic selection (GS)”, and “GS”) and Boolean operators were used to query the database. The literature on “grain yield” was very extensive in bread wheat and covered different branches of knowledge [37,38]. However, by restricting the search to durum wheat, the number of published papers was drastically reduced, representing only 40% of all those published (Figure 2).

**Figure 3:**
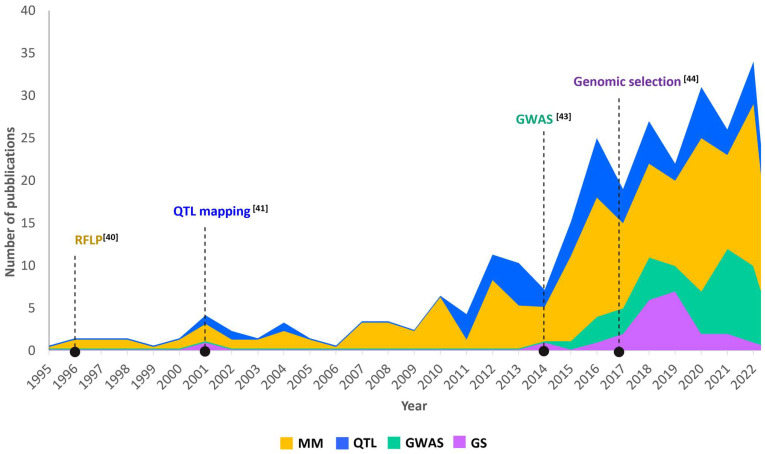
Number of publications in which yield and yield components traits were investigated by using different genomic approaches in wheat. MM = molecular markers, complete query = “durum wheat” AND “yield” AND (“RFLP” or “SSR” or “AFLP” or “DArT” or “SNP”); QTL = QTL mapping, complete query = “durum wheat” AND “yield” AND “QTL mapping”; GWAS = genome-wide association mapping, complete query = “durum wheat” AND “yield” AND “GWAS”; GS = genomic selection, complete query = “durum wheat” AND “yield” AND “genomic selection”. Data referred to 2022.

Effectively, using “yield” and “genetic or genomic” as keywords, the results showed the largest number of papers in bread (8686) and in durum wheat (1427). Subsequently, for defining the number of papers that used molecular markers to study yield and yield components, keywords such as “durum wheat”, “yield”, “RFLP”, “SSR”, “AFLP”, “DArT”, and “SNP” were used and, only 148 studies resulted that were published from 1995 to 2021 (Figure 3).

Until 1995, studies on yield in durum wheat were based on the relief of morphological traits. Genetic aspects were dealt with in comparison with the more advanced results in bread wheat. In 1996, the first paper that aimed to study QTL controlling grain yield in durum wheat was performed by using RFLP markers on a segregant population derived by crossing the durum wheat cv. “Messapia” and the dicoccoides accession “MG4343” [40]. However, only in 2001 the keyword “QTL mapping” was related to “durum wheat” and “yield components” [41] (Figure 3). Since then, an average of 2.5 papers were published per year until 2014, when the first SNP array was released [42]. The advance in genomic technologies and the release of the 90K Wheat Infinium iSelect SNP array overlapped with the first GWA study for yield and yield components in durum wheat [43] and with a significant increase in new papers. A few years later, a study by Fiedler et al. [44] concerning the genomic selection for grain yield was published (Figure 3), opening the way to a new generation of studies. In 2022, the number of papers relating to genomic approaches in durum wheat for yield was 34 (data referred to 2022).

## 3. QTL Mapping Strategies for Yield and Its Components in Durum Wheat

The most powerful and used strategy for breaking down the components of grain yield was and remains QTL mapping, through linkage map approaches as well as GWA mapping studies [45,46].

QTL mapping is based on the identification of molecular markers associated with a given phenotypic trait in a segregating population and/or germplasm collection, thus allowing the positioning of QTL within genetic or, more recently, physical maps. The identification of molecular markers for specific QTLs can help to improve and accelerate breeding programs. The advance in genomic strategies and the availability of a large number of SNP markers, distributed along the genome, has allowed researchers to accurately characterize and discover loci underlying yield and its components. In addition, the recent sequencing of the durum wheat genome [47] has given a strong impetus to genetic studies on yield by narrowing the associated genomic regions and favoring the study of the relationships between candidate genes within the QTL.

Herein, we proposed an extensive but non exhaustive overview of loci associated with grain yield, merging the information available in the literature mainly for durum wheat but also for bread wheat. Based on a reductionist approach, we examined the QTLs discovered for grain yield moving from its components as reported in Figure 1.

### 3.1. Genomic Regions Associated with Grain Number (GN)

**Figure 4:**
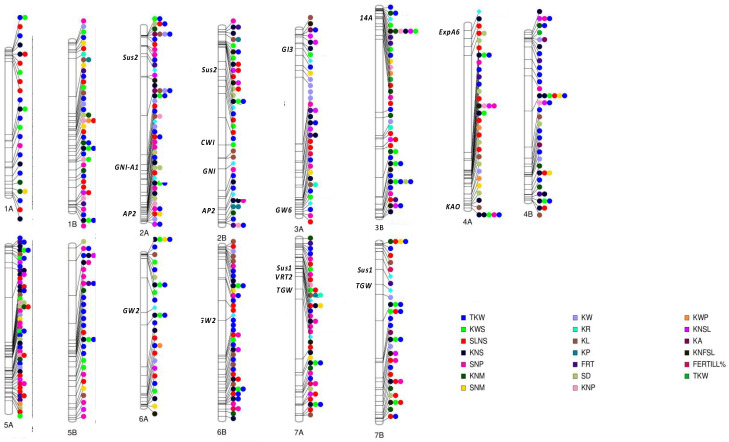
Schematic representation of identified Quantitative Trait Loci for yield and yield components on chromosomes of durum wheat genome. The ideogram of all 14 chromosomes is plotted, and lines correspond to the putative base-pair location of each QTL. Each color represents a different phenotype: thousand kernel weight (TWK), grain yield/spike (KWS), spikelets/spike (SLNS), kernels/spike (KNS), spikes/plant (SNP), kernels/m^2^ (KNM), spikes/m^2^ (SNM), kernel width (KW), kernel roundness (KR), kernel length (KL), kernel perimeter (KP), spike fertility (FRT), spike density (SD), grain yield/plant (KWP), kernels/plant (KNP), kernels/fertile spikelet (KNSL), percentage of fertile tillers (Fertill %). The putative physical position of causative genes is also reported. The plot was drawn using PhenoGram online tool (https://ritchielab.org/software/phenogram-downloads, 28 July 2023).

Studies on bread and durum wheat yield have shown that increases in grain yield are always accompanied by an increase in GN, suggesting that boosting yield potential may require additional improvements of subcomponents’ related traits [34,48]. Since, the number of plants/m^2^ (PD) is basically determined by the sowing rate and seed quality (i.e., rate of germination), the genetic interest was oriented mainly to the remaining GN subcomponents such as spikes/m^2^ (SNM), spikelets/spike (SLNS), and kernels/spike (KNS). Spikes/m^2^ (SNM) is one of the most important yield components and is directly affected by fertile tiller number/plant (FTN). The increase in FTN generally enhances yield potential over a range of environmental conditions [49], and, among all the subcomponents of grain yield, it is the one with the lowest heritability values [9] and a great compensation capacity [49]. Indeed, several QTLs that control tillering have been described in wheat, generally in bread wheat [20,49,50,51,52,53,54,55,56,57,58] but also in durum wheat. Giunta et al. [9], analyzing the physiological association between phenology and tillering capacity in durum wheat, identified a total of 33 QTLs spread over several chromosomes according to previous studies [49,53,54,56]. Despite the greater stability, QTLs for other subcomponent traits have been identified on almost all chromosomes by using both linkage maps and GWA approaches (Figure 4) [6,11,59]. Mangini et al. [11] reported eight QTLs for kernels/spike in 233 genotypes belonging to modern and old durum cultivars, landraces, domesticated and wild types. Most of the QTLs for KNS were found in different genomic regions from those reported for TKW, indicating that KNS and TKW are genetically controlled independently by each other. Out of eight KNS QTLs, three were associated with significant increases in grain yield/spike (KWS) [11]. In contrast to KNS, it has been shown that most of the QTLs for kernels/m^2^ (KNM) co-localized with those for TKW, and generally showed an opposite additive effect on each trait. Sukumaran et al. [8] identified two homoeologous loci with an opposite effect on chromosome 2 associated with GY, TKW, and KNM in a durum wheat population grown under yield potential. The locus on chromosome 2A (61–70 cM) was associated with TKW and KNM, whereas the locus on chromosome 2B (78–82 cM) was related to TKW, GY, and KNM. Soriano et al. [7] also disclosed several QTLs for kernel/m^2^ through the GWAS approach using a collection of 172 durum wheat landraces representative of the genetic diversity of ancient local durum varieties from the Mediterranean basin. These QTLs were distributed on 7 out of the 14 chromosomes (except 2A, 2B, 3A, 4A, and 6A), and they explained a total phenotypic variation ranging between 3.3% and 18.8%. The increase in KNS was also attributed to spike fertility (FRT) [60]. In a recent study by Anuarbek and colleagues [61], 83 marker–trait associations (MTAs) have been identified in two contrasting environments through the GWA approach for many traits related to yield components, including the percentage of fertile tillers (Fertill %) and kernels/spike. By comparing the identified MTAs with those previously reported in GWAS for durum wheat, Anuarbek and colleagues [62] suggested that 38 MTAs were presumably novel, whereas the remaining MTAs overlapped with those previously published in the literature, confirming the validity of their results. Other QTLs, distributed on all durum wheat chromosomes, have been also disclosed for Fertill % [9] and FRT [62]. The former identified genomic regions on chromosomes 3A, 5A, 6B, 7A, and 7B by using 98 genotypes derived from crossing “Ofanto” × “Cappelli” [9], whereas the latter found QTLs on all chromosomes, except 1A, 6A, and 6B using two mapping populations from “Kofa” × “UC1113” and “KU7309” × “KU8736A” [62,63]. However, although KNS and FRT are positively correlated with each other, both are negatively correlated with GW, inducing a decrease for this latter trait [64]. Besides the aforementioned regions, other QTLs were related to spikes/plant (SNP) [5,9,65] and spikes/m^2^ (SNM) [7,66,67] by using RIL populations developed from different crosses such as “Ofanto” × “Cappelli”, “Kofa” × “UC1113”, “H52” × “Langdon”, “Kofa × Svevo”, “Svevo × Zavitan”, and germplasm collections. Most of the QTLs described above with both moderate and major effects (>20% of the phenotypic variation) were included in a consensus map developed by Maccaferri et al. [68].

### 3.2. Genomic Regions Associated with Grain Weight (GW)

Although grain weight with respect to the grain number is stably inherited [23], it is also composed of multiple subcomponents, including grain morphometric parameters (i.e., kernel length, KL and kernel width, KW) [69,70]. More than 220 QTLs were identified on all chromosomes, most of which were related to TKW (about 144). Numerous QTLs related to GW were detected using bi-parental populations, e.g., DH, BILs, RILs, and F2 populations developed from durum and dicoccoides crosses or tetraploid wheat collections [10,11,41,59,71,72,73,74,75,76,77,78]. Probably since GW was mainly the result of wheat evolution under domestication [14,79], the use of segregating populations derived from crosses between wild and domesticated wheat was preferred—for example, Lin et al. [80].

Stable QTLs for GW and TKW were identified on chromosomes 1B and 2A. The “IWB20542” marker identified a QTL region controlling TKW on chromosome 1B and explained more than 20% of phenotypic variance [66,73,76], whereas a QTL on chromosome 2A explained, on average, ~12% of phenotypic variation [4,10,53,74,75,77,81,82], and it was mapped near the photoperiod sensitivity gene *Ppd-A1* [4,10,11,75]. However, the *Ppd-A1* gene appears to have a negative pleiotropic effect on TKW and grain size, whereas it has a positive effect on other yield components such as KNM [9,10,59]. Ji et al. [83] also identified major QTL for grain size and weight on chromosome 4A and 6A in bread wheat, and results were validated through Kompetitive Allele Specific PCR (KASP) markers. Among the identified regions, the authors suggested that QTL on chromosome 4A was new, and its associated SNP marker can be used in wheat breeding [84].

TKW was affected also by the pleiotropic effect of the dwarfing gene; indeed, QTLs for GW and TKW colocalized with *Rht-B1* gene on chromosome 4B, explaining more than 20% of the phenotypic variability [7,74,85].

QTLs for TKW were also found on 6B, consisting of ~10% of phenotypic variance [7,10,11,72,85]. Other QTLs were detected on chromosome 3B, 5B, 6A, 7A, and 7B by using both linkage maps and GWA approaches [6,11,59,86,87].

As regards the QTLs for kernel area, kernel width, and kernel length, the SNP markers associated exhibited a R^2^ < 10%, suggesting that the kernel-related traits are controlled by many loci with minor effects in durum wheat [82]. Stable QTLs for kernel area were detected on chromosomes 2A, 3A, 4B, 5A, 5B, and 6B [10,82], out of which, the one on chromosome 2A explained more than 40% of phenotypic variance and co-localized with the same region of TKW [10]. QTLs controlling kernel width were distributed on several chromosomes (1B, 2A, 4B, 6B, and 7B), and they explained 5.1–12.1% of the phenotypic variance [10,82]. Russo et al. [76] reported six QTL on chromosomes 1B, 2B, 3A, 3B, 4B, and 7A and two QTL on chromosomes 3B and 4B for kernel morphology traits.

Generally, TKW was always positively and significantly correlated to kernel area, kernel width, and kernel length; indeed, several QTL regions overlapped. In particular, the marker BE500291_5_A_37 identified a stable SNP for TKW, kernel area, and kernel length [82].

Although QTL mapping analysis is a powerful tool in agriculture because it provides knowledge of the desired QTL for complex traits and can be used in breeding programs, it is strongly influenced by many factors, including the choice of markers and their density, the type and size of mapping population, and environmental conditions [45]. To overcome this limitation, meta-QTL (MQTL) analysis has been proposed as a powerful and robust approach for genetic dissection of complex quantitative traits [13]. MQTL analysis combines the QTL results from independent studies (both linkage mapping and GWA) and refines the QTL position on the consensus maps, increasing selection accuracy and efficiency, thus enhancing genetic gains in plant breeding programs [7].

MQTL analysis was also successfully employed to detect consensus QTL regions in wheat [7,13,60,88,89,90,91,92,93], especially for complex traits such as yield and yield components [7,89,93,94].

In durum wheat, to obtain an overview of the QTL distribution, the consensus map developed by Maccaferri et al. [95] was used for QTL projection based on the homothetic approach described by Chardon et al. [96]. The latest work mapped 2230 initial QTLs of yield and its components from 119 independent QTL studies to the wheat reference genome, resulting in 145 MQTLs distributed across all chromosomes [93].

As far as concerns durum wheat, recently, a rare allele of growth-regulating factor 4 (*GRF4*) was found associated on chromosome 6A with larger grains using MQTL analysis [89]. Another work projected 477 unique QTLs for yield formation, crop phenology, and crop biomass against a durum wheat consensus map, identifying 71 meta-QTLs involved in yield, yield components, and phenological development on 1B, 2B, 3A, 3B, 4A, 5A, 5B, 7A, and 7B chromosomes [7]. However, the 61% of QTLs account for less than 10% of the phenotypic variance, confirming the high genetic complexity of the traits analyzed.

A recent work used 2852 major QTLs of 8998 QTLs available for yield and related traits for meta-analysis, identifying 141 meta-QTLs, out of which 13 were breeder’s MQTLs for use in MAS for grain yield improvement in wheat [97]. Similarly, Ma and colleagues [15] used 1103 original QTLs from the 34 studies to refine 58 MQTLs that can be explored for breeding purposes.

Based on this evidence, it was clear that the adoption of the reductionist approach, although useful to simplify the identification of the QTLs associated with the yield through its subcomponents, remains equally complex, making the integration of QTLs into breeding programs very difficult.

## 4. Genes Affecting Yield and Its Components in Durum Wheat

The search for candidate genes within the confidence interval of the major QTLs identified in the previous studies often co-mapped to well-known adaptive genes (i.e., *Ppd-1*, *Vrn-1*, and *Rht-1*). In addition to the well-known pleiotropic effect exerted by the *Rht-1* gene on spike fertility (i.e., number of seeds per spike and number of seeds per spikelet), also *Ppd* genes played a key role in the formation of the terminal spikelet and GN [98,99,100]. On the contrary, *Vrn* genes did not show a clear effect on the number of total spikelets [9,51] but significantly influenced the tillering capacity not only in bread [50] but also in durum wheat [9]. Therefore, identifying the specific genetic determinants underlying the variation in yield and its components was very difficult. The manipulation of environmental conditions provided the opportunity for each group of genes to be expressed without confounding effects of the others, and for the definition of the interactions of each QTL with the environment [9]. Certainly, the recent release of the “Svevo” durum wheat genome has made it possible to increase the accuracy of the detection of QTLs, to identify candidate genes within the QTLs, and to identify pleiotropic effects between them.

To date, numerous studies have identified candidate genes in cereals, and some of these have been cloned in rice [101,102], barley [103], and maize [104,105], and some genes associated with GY, GN, and GW were also identified in bread wheat [56,106,107,108,109,110,111,112,113,114,115,116]. Clues regarding the identification of ortholog genes in durum wheat are still scant. For this reason, here, by using the aminoacidic sequence of candidate genes identified in bread wheat and other species, their physical position was projected on durum wheat pseudomolecules (Figure 4).

Among candidate genes associated with grain yield, Marcotuli et al. [117] reported an *APETALA-2-like* (*TaAP2*), a *GIGANTEA 3* (*TaGI3*), and a 14-3-3 protein (Ta14A). *TaAP2* on chromosome 2B played a central role in the transition phase from vegetative to reproductive growth [118]. *TaGI3*, on chromosome 3A, was known to affect the photoperiod pathway and flowering promotion in wheat [119]. The 14-3-3 protein (Ta14A), on chromosome 3B, binds transcription factors and signaling proteins, participating in the regulation of kernel and plant development, but it is also involved in biotic and abiotic stress responses [120].

Among the most important genes related to grain number, the locus *Grain Number Increase 1* (*GNI1*) encodes a homeodomain leucine zipper class I (*HD-Zip I*) transcription factor, which is most strongly active during the growth and development of the apical florets and the rachilla. In 2020, Sakuma and colleagues [121] disclosed natural variation within the *GNI-A1* locus by resequencing the allele in 72 tetraploid wheats, including both wild and domesticated emmer and durum wheat. Among the identified haplotypes, the 105Y variant featured a significantly higher number of kernels/fertile spikelet (KNSL) in durum wheat entries than in emmer wheat entries, when plants were grown in three different environments. In addition to the above examples, Sun et al. [82] annotated a total of 54 candidate genes for kernel-related traits in a worldwide collection of durum wheat germplasm. In this study, the authors reported that the SNPs “BE500291_5_A_37” on chromosome 5A and “BF474023_3_A_Y_425” on chromosome 3A, which overlapped with *1-acyl-sn-glycerol-3-phosphate acyltransferase* (*PLS1*) and *Abscisic Acid Insensitive-like 1* gene (*ABIL1*), respectively, could play an important role in the kernel development of durum wheat. Adamsky et al. [122] identified through a map-based cloning approach a Vegetative to *Reproductive Transition 2* gene (*VRT2*), which encodes a *MADS-box* transcription factor as the gene responsible for the longer glumes and grains in Polish wheat. The authors reported that a mutation in intron-1 resulted in ectopic expression of the *VRT-A2* allele. Transgenic lines of hexaploidy wheat carrying the *T. polonicum VRT-A2* allele were highly correlated with spike, glume, grain, and floral organ length, highlighting how changes in expression profiles can affect agronomic traits in a dosage-dependent manner in polyploid crops. At the same time, Li and colleagues [123] reported a *Squamosa* gene as a negative regulator of *VRT2* as well as a *Short Vegetative Phase 1* gene (*SVP1*) and *SVP3*, suggesting that the manipulation of these genes can contribute to engineering spike architectures, thus improving wheat productivity and yield. Recently, Dong et al. [124] cloned the *tiller number 1* (*TN1*) gene via map-based cloning in wheat, highlighting that the inhibition of tiller bud outgrowth in the tn1 mutant may be caused by enhanced ABA accumulation and ABA signaling in the tiller buds. Based on their findings, the authors proposed a working model on *TN1* in regulating wheat tillering.

As regards candidate genes associated with grain weight, the *Grain Weight 2* (*TaGW2*) locus encoding a RING-type protein with E3 ubiquitin ligase activity was the most studied. It has been shown in both bread and durum wheat that loss-of-function mutations in the coding sequence of *TaGW2* resulted in enhanced KW and GW [106,125]. In particular, a splice acceptor site mutation in *GW2-A1* increased thousand grain weight through wider and longer grains. Later, in 2016, Simmonds and colleagues confirmed the results in both tetraploid and hexaploid wheat, reporting that null mutants in *TaGW2-A1* increased not only TKW but also kernel width (up to 2.8%) and kernel length (up to 2.1%). Specifically in durum wheat, Sestili et al. [126] reduced the abundance of the same gene in the cultivar “Svevo” through the RNAi approach, obtaining an increase in the kernel width from 4 to 13%. Another interesting gene related to grain weight in bread wheat is the *TaTGW6-A1* gene, a direct ortholog of rice *TGW6*, which has been reported to encode a protein with indole-3-acetic acid (IAA)-glucose hydrolase activity that influenced the grain weight [127]. Hu and colleagues [128] reported three different alleles at *TaTGW6* locus in bread wheat (*TaTGW6-a*, *TaTGW6-b*, and *TaGW6-c*). *TaTGW6-b* had a 6-bp InDel at position 170 downstream of the initiation codon, whereas *TaTGW6-c* was the null mutant. Both alleles significantly increased grain size and weight compared to the *TaTGW6-a* allele, although *TaTGW6-b* and *TaTGW6-c* had a low frequency distribution in modern wheat varieties. Recently, the *WRKY* transcription factor, *TaGSNE*, was significantly related to an increase in TKW and spikelet number per spike, overcoming the tradeoff between grain size and grain number. The advantageous haplotypes have been suggested in MAS breeding programs for selecting high-yielding varieties [129]. Other genes such as a *serine carboxypeptidase*, *TaGS5*, an *E3 ligase*, *TaGW2*, a *ubiquitin receptor*, *TaDA1*, a *cytokinin oxidase/dehydrogenase*, *TaCKX6*, an *expansin*, *TaExp6*, a *F-Box* gene, *WAPO1*, a transcription factor, *TaGNI*, and *KLW1* have been identified as potential candidate genes affecting grain weight and grain number in wheat [130,131].

Genes involved in starch and sucrose metabolism pathways were also shown to affect grain weight. As an example, *Sucrose Synthase 1* gene (*TaSus1*) was cloned and characterized in bread wheat. It has been shown that the favored haplotype (TaSus1-7A-Hap-5) caused by a mutation that induces lower enzyme activity was closely correlated with higher TKW in two different environments [132]. *TaSus2* was also isolated and mapped in wheat by Jiang et al. [133]. *TaSus2* was mapped on chromosome 2B in a region where two MQTLs for grain weight (MQTL15 and 17) were found by Soriano et al. [7].

The *cell wall invertase* gene (*CWI*) is also a critical enzyme for sink tissue development and carbon partition and showed a high association with GW [134]. Ma and colleagues [134] characterized the full-length genomic region of CWI on chromosome 2A of wheat near an MQT (MQTL11) identified by Soriano et al. [7]. Rustgi et al. [135], using the rice genome, identified two orthologous genes (*CKX2* and *GID2-like*) on chromosome 3A of wheat spanning QTLs for yield. Later, Soriano et al. [7] suggested that the location of these two genes may correspond to their MQTL22, 23, and 24, although only the last two MQTLs were associated with GY, GW, and spikes/m^2^. A novel gene *TaKAO-4A* (*TraesCS4A02G460100*) on chromosome 4A was also found to be significantly correlated with grain size, and it was validated through the development of a CAPS marker, thus improving the knowledge for the identification and combination of these important QTLs or candidate genes in wheat high-yield breeding [93]. Besides the above genes, another way to regulate grain weight is through expansin manipulation, which can lead to changes in growth and development [136,137,138]. Calderini et al. [139] described how the overexpression of an *α-expansin* in early-developing wheat seeds leads to a significant increase in grain size and, in consequence, grain weight without a negative effect on grain number. The overexpression of the functional orthologue of *OsBG1* in wheat (*TaBG1*) confirmed previous findings in rice by increasing the seed size, but it did not increase yield, and it limited the concentration of essential elements as well as potentially lowered protein content [140]. Recently, a study demonstrated that the overexpression of the *cytochrome P450 monooxygenase KLUH/CYP78A5* significantly increased seed size and weight but not grain yield. However, natural variations in the promoter of *TaCYP78A5-2A* contributed to TGW and grain yield per plant of wheat; a functional marker of *TaCYP78A5* haplotype Ap-HapII has been developed for MAS in wheat yield improvement [141].

## 5. A Holistic Approach to Studying Yield and Its Components in Post-Genomic Era

Brinton and Uauy [23] argued that a reductionist approach should not be confused with the study of subcomponents in isolation. Indeed, the knowledge of the mechanisms that control yield-related architectural traits as well as the regulation of individual genes that control the single yield components will allow us to better evaluate the negative correlations between the yield components with the aim to dissociate them [142]. In fact, this approach, as we highlighted in the previous paragraph, led to the identification of target genes for single components and their functional validation. Despite this, the tradeoff between GN and GW has not been resolved, and although the experimental field trials validation, in rare cases, was effective, it depended on the genetic background with different compensatory effects [143].

The genetic complexity of the yield trait and its components, together with the difficulty of conducting precise phenotyping for these traits, also makes it difficult to develop specific genetic stocks (i.e., near-isogenic lines, mutants) suitable for the candidate genes identification and validation. In addition, the polyploid nature of wheat does not facilitate this task due to the masking effect of the single genes, which often occurs in this species because of the copies present on the homoeologous chromosomes.

With the rapid advances in sequencing and bioinformatic technologies, innovative and accelerated strategies based on targeted editing approach will allow a better manipulation of the genes and, consequently, an acceleration in the understanding of the gene interactions existing between the components of the yield [144,145]. These strategies include revealing the biological function of genes by characterizing their corresponding mutants, identifying the gene responsible for an interesting phenotype by gene cloning, and verifying the function of a target gene by transforming the candidate wild-type gene into the corresponding wheat mutant. Haplotype-based association mapping, mutational mapping (MutMap), mutant chromosome sequencing (MutChromSeq), targeted chromosome-based cloning via long-range assembly (TACCA), and mutagenesis resistance gene enrichment and sequencing (MutRenSeq) have been employed to overcome the limitations of traditional gene cloning methods [146]. The haplotype-based AM approach was suggested as an efficient method for investigating the genetic basis of traits of interest in durum wheat by detecting more loci [147], capturing epistatic interactions, and reducing type I error rate [148,149,150]. N’Diaye and colleagues [147] identified 21 haplotype loci associated with multiple traits. Among them, hap_4B_1 explained phenotypic variance ranging between 16.6% and 20.6% for pigment loss and dough extensibility. Similarly, hap_2B_9 was associated with variation in protein content, explaining a phenotypic variance up to 18%. MutMap is another rapid method based on mutagenesis, gene mapping, and whole-genome resequencing. Schoen et al. [151], by crossing the low tillering mutant with the Jagger wild-type plant, characterized a novel tillering number gene using the MutMap approach. However, this approach is more suitable for small genomes such as rice, where it has been used to identify the genomic positions of genes controlling agronomically important traits. By contrast, MutChromSeq is a good choice for gene cloning from large genomes. The approach combines mutagenesis, the reduction of genome complexity by chromosome sorting, and high-throughput sequencing, opening a new way to identify candidate genes by comparing the wild-type and mutant chromosomes. An example is the wheat Pm2 gene, which has been analyzed using this method [152]. TACCA also enables rapid gene cloning from complex polyploid genomes. Using this method, the wheat leaf rust resistance gene Lr22a was isolated in bread wheat [153].

In the expectation of better exploiting the tools offered by genomics and sequencing of wheat genomes to solve the potential tradeoff between the multiple components of grain yield, genomic selection (GS) represents the most effective tool to accelerate genetic gain for complex traits such as yield and its components [154,155,156,157,158]. In maize, GS was reported to achieve up to threefold annual genetic gain improvement when compared to MAS, due to a more efficient accounting of trait-associated QTL, faster selection cycles, and lower phenotyping costs [159,160]. The concept on which genomic selection is based consists of merging all available marker information simultaneously into a model to predict the breeding values of breeding progenies for selection [161,162]. Several factors affect the genomic prediction accuracy in GS, as well as training population size, trait complexity, and marker density [163].

Most of the genomic selection studies have been carried out in bread wheat [90,164,165,166,167,168], whereas GS remains largely unexplored in durum wheat [169,170,171,172]. However, the need to develop high-yielding and climate-resilient varieties is prompting the use of the GS approach to improve grain yield even in durum wheat. In this regard, Fiedler et al. [44] tested different GS models, achieving values between 0.27 and 0.66 for the prediction of breeding for five different traits associated with grain quality. It is noteworthy that the information of previously identified QTLs as well as the information about the allelic profile of known genes such as *Vrn*, *Ppd*, and *Rht* can now be included in genomic prediction models to increase their accuracy [173]. Similarly, Haile et al. [170] reported an accuracy ranging between 0.5 and 0.8 for traits associated with yield and grain quality in durum wheat. Recently, Zaïm et al. [67] obtained a higher precision in the prediction for grain yield and TKW. When the QTL-underlying markers were used in the model, the accuracy for the former trait increased from 0.37 to 0.54, whereas that for the latter increased from 0.30 to 0.54. Finally, a combined approach between associative mapping and genomic selection, together with the availability of high-density SNP array and the reference genome, will be pivotal for increasing genetic gains for yield and its components in future durum wheat breeding programs. Probably, as suggested by some authors attempting to simultaneously improve multiple traits (i.e., grain yield and protein content) using genomic selection [170,174], it could be useful to combine different breeding strategies in the form of selection indices and improve the final yield by synergistically exploiting the single yield components.

## 6. Conclusions and Future Perspectives

The identification of genes and/or chromosomal regions controlling the grain yield in durum wheat is still a challenge, mainly due to the polyploidy level of this species and the quantitative nature of the traits under investigation. To overcome this issue, NGS and high-throughput phenotyping platforms, which combine more precision in trait detection and big data generation by means of high-performing computing technologies, will revolutionize plant breeding, providing a great opportunity for capturing genetic variation that can be used in breeding programs.

If until today, classical breeding, adopting a reductionist approach, has represented the key to success in varietal development through the definition of a priori plant ideotype, the paradigm shift achieved through genomic selection and genome editing represents the turning point for yield improvement solving the complexity of the system. With the huge amount of wheat genome sequence data being rapidly generated, GS provides a valuable tool for statistically predicting the value of a given genotype in selection by modeling the optimal allelic combinations across the whole genome that they maximize the phenotypic potential of an individual under a given environment [169,175].

Since many genes have been identified as putatively associated with yield and its components, some of them might be validated through genome-editing technologies (CRISPR/CAS9), which currently have revolutionized the plant research field and reveal the great potential for its use in crop improvement. In addition, genome editing promises to create new alleles, correct defective alleles, and/or to a certain degree, pyramid compatible alleles across the genome to achieve the desired phenotypes, finding a tradeoff solution for improving grain yield in durum wheat.

To conclude, the perspectives on how -omics technologies can exploit the opportunity to create new phenotypes, as well as what could be achieved through the more reductionist approach of functional genomics, are represented by genomic modeling. The challenge, however, remains to transfer the enormous amount of information produced by the international scientific community on wheat bread to durum wheat.

## Data Availability

No new data were created or analyzed in this study. Data sharing is not applicable to this article.
